# Experimental Evaluation of Chitosan Membrane and Collagen Sponge (TachoSil®) as Sealants in Cardiovascular Surgery

**DOI:** 10.21470/1678-9741-2021-0432

**Published:** 2022

**Authors:** Renan Nalin dos Santos, Guinea Brasil Camargo Cardoso, Marcelo Rodrigues Cunha, Evaldo Marchi, Marcus V. H Carvalho

**Affiliations:** 1 Department of Surgery, Faculdade de Medicina de Jundiaí, Jundiaí, São Paulo, Brazil.; 2 Laboratory of Biomechanics, Faculdade de Engenharia Mecânica, Universidade Estadual de Campinas, Campinas, São Paulo, Brazil.; 3 Department of Morphology and Pathology, Faculdade de Medicina de Jundiaí, Jundiaí, São Paulo, Brazil.

**Keywords:** Femoral Artery, Fibrin Tissue Adhesive, Ca, Hemostatics, Fibrinogen, Catheterization, Cardiovascular Surgical Procedures

## Abstract

**Introduction:**

The objectives of this study are to experimentally evaluate the haemostatic effects of two organic substances, a membrane of chitosan and a collagen sponge coated with thrombin and human fibrinogen (TachoSil®), in sealing 7-0 needle stitches holes on the femoral arteries of rats as well as to evaluate local histological reactions.

**Methods:**

Twenty-four rats were included, and four holes were made in each common femoral artery. In the control group, haemostasis was achieved only by compression with gauze sponge; and in the two other groups, haemostasis was achieved with application of one of these two substances.

**Results:**

Membrane of chitosan and TachoSil® showed a power to reduce the time to achieve haemostasis compared with the control group (P=0.001), and the haemostatic effects of these two substances were comparable. There was lower blood loss in the groups where these two substances were used when compared with the control group, but no difference was found comparing the two substances.

**Conclusion:**

The use of these sealants did not promote more adhesion or local histological reactions when compared to the control group. Since chitosan is easy to find in nature, has a positive effect to promote haemostasis, and did not bring considerable local reactions, it might be used as a sealant in cardiovascular surgery.

**Table:** Abbreviations, Acronyms & Symbols

CG	= Control group
ChG	= Chitosan group
LV	= Lumen of the vessel
TA	= Tunica adventitia
TBV	= Total blood volume
TG	= TachoSil® group
TI	= Tunica intima
TM	= Tunica media
VW	= Vessel walls

## INTRODUCTION

Vascular sutures are not always effective, particularly in those patients who use anticoagulants or antiplatelet agents. Sealants can curb or decrease bleeding on the suture line of blood vessels, and they can be substances of organic or synthetic origin, the former having less potential to cause inflammation^[1]^.

Chitosan, a mucopolysaccharide derived from chitin by deacetylation process, is the second most abundant polymer in nature and is considered a natural fibre with high biomedical potential due to its biocompatibility, biodegradability, and absence of toxicity^[2]^.

The aim of the present study was to experimentally determine the effects of two organic materials, a membrane composed of chitosan and a collagen sponge coated with thrombin and human fibrinogen (TachoSil®), in repairing the femoral artery of rats. The haemostatic capacity as well as macroscopic and local histological reactions were checked, comparing the referred effects between the chitosan membrane and collagen sponge with the control group (CG).

## METHODS

This study was approved by the Ethics Committee for Animal Research of the Faculdade de Medicina de Jundiaí (protocol 262/2016), which follows the recommendations of the National Council for Animal Experimentation of Brazil (or CONCEA).

The polymeric chitosan membranes were manufactured and supplied by the Polymer Laboratory at the Faculdade de Engenharia Mecânica of the Universidade Estadual de Campinas, using Sigma-Aldrich® polymers (Merck KGaA, Darmstadt, Germany).

TachoSil® (Takeda Austria Gmb) is a sponge made of collagen coated with 5.5 mg of human fibrinogen per cm^2^ and 2.0 I.U. human thrombin per cm^2^.

Twenty-four rats (*Rattus norvegicus*, Wistar) were used, with an average age of 26 weeks and weight between 250 g and 350 g. The animals received balanced feed (Purina®), water *ad libitum* in a controlled temperature environment (23±1°C), and a 12/12 hours light/dark cycle.

The 24 animals were randomly assigned to three groups, each of which was subdivided into two subgroups. The animals in the first subgroup were euthanized on the 7^th^ postoperative day, and the animals in the second subgroup were euthanized on the 21^st^ postoperative day. Four perforations were made in both femoral arteries of each animal, and haemostasis was obtained as follows:

- Control Group (CG), n=8: compression with sterile gauze.
- Chitosan group (ChG), n=8: allocation of the chitosan-based membrane around each femoral artery, followed by compression with sterile gauze.
- TachoSil® group (TG), n=8: allocation of a TachoSil® sponge film around each femoral artery, followed by compression with sterile gauze.

### Surgical Procedure

The animals were anesthetized with ketamine Francotar® solution (Sespo Ind. E Com., Jacareí, Brazil) and 2% xylazine hydrochloride Xilazin (Virbac Brasil Ind. E Com., São Paulo, Brazil) — proportion 2:1, dose 0.15 ml/100 grams of body weight, intramuscularly. The experiments took place under sterile conditions. The rats were placed in the supine position, and trichotomy antisepsis was performed in the femoral regions, bilaterally. The procedures reported below were done first on the right and then on the left side. Small incisions (0.5 cm) were made in the right and left inguinal regions, followed by dissection and isolation of the common femoral artery. Subsequently, two transfixing perforations were made with stainless steel needle of polypropylene 7.0 non-absorbable monofilament suture thread (PolySuture®, S. Sebastião do Paraíso, Brazil), resulting in four perforations in each common femoral artery. In the CG, compression was made with sterile gauze until the bleeding stopped, and in the other groups, compression with gauze was performed after the application of predetermined material (chitosan or TachoSil® membrane) around the femoral arteries in each animal.

Since both femoral arteries were injured in each animal, each group had 16 femoral arterial lesions.

The gauzes used on the femoral arteries were weighed before and after compression, and the values were recorded. The time until complete bleeding suppression was also counted and noted.

After the procedure, the incisions were closed with nylon 4.0 monofilament surgical suture (Shalon®, Goiânia, Brazil). Each animal received injection of 0.1 mL/100 g weight dose of the veterinary antibiotic Pentabiotic® for small size animals (Fort Dodge, Campinas, Brazil), via gluteal intramuscular. All animals were kept under observation with artificial heating until awakening from anesthesia and sent to the vivarium. It was made available for the animals to drink paracetamol solution at a dosage of 20 mg/kg of weight, diluted in water until the date of euthanasia.

The animals were kept isolated in separate cages, with continuous observation, and received common food and water *ad libitum*. The wounds were inspected in order to check the integrity, possible infections, and the aspect of healing.

On the 7^th^ postoperative day, animals from subgroups CG7, ChG7, and TG7 were euthanized (lethal dose of sodium thiopental, 100 mg/kg, intraperitoneally) to check for scar reactions (beginning of the proliferative phase of the healing process). The animals from subgroups CG21, ChG21, and TG21 were euthanized on the 21^st^ postoperative day to study healing reactions (proliferative and maturation phases of the healing process).

- CG: four animals euthanized at the 7^th^ day, and four animals at the 21^st^ day.
- ChG: four animals euthanized at the 7^th^ day, and four animals at the 21^st^ day.
- TG: four animals euthanized at the 7^th^ day, and four animals at the 21^st^ day.

### Analysis of Haemostatic Capacity

The haemostatic capacity of the membranes was assessed by the time elapsed until the haemorrhage was suppressed (time to haemostasis) and the amount of blood absorbed in the gauzes.

Considering that the total blood volume (TBV) of *Rattus norvegicus* is 6.4% of its weight, the TBV of each animal was first calculated. Subsequently, the volume of blood lost by each animal was calculated. As the amount of blood lost was measured in grams by the weight of the gauze, it was necessary to convert the grams into milliliters (mL) considering the blood density as the same as that of water, taking into account that previous research demonstrates this method^[3]^.

### Macroscopic Analysis

After euthanasia, the surgical wound was reopened, and the adhesions of the femoral arteries with adjacent tissues were analysed and classified according to the criteria of Knightly et al.^[4]^.The adhesion analysis was performed only in the groups in which euthanasia was performed at 21 days after the procedure (CG21, ChG21, and TG21), and adhesions were classified as: 0, without adhesions; 1, light adhesions and easy separation of tissues; 2, firm adhesions with moderate difficulty in separating the tissues; 3, very firm adhesions requiring difficult dissection for tissue separation.

### Microscopic Analysis

**Fig. 1:**
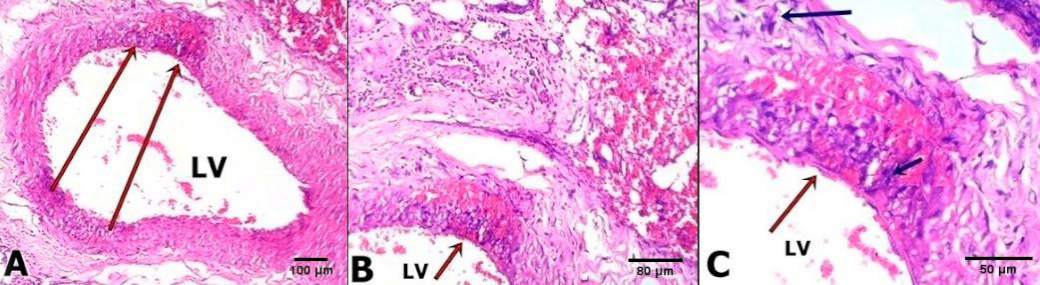
Photomicrographs of the region of surgical specimens extracted from samples 7 days after the operation, stained with HE. A, B and C belonging to the Control Group (GC). Note in Figure A, B and C, a greater amount of red blood cells in the vascular wall, and adjacent regions beyond the lumen of the vessel (LV). In A (10x magnification), the long red arrows point to the route of vascular injury (tunics with infiltrates of red blood cells and leukocytes, in addition to greater epithelial density). B (20x magnification) and C (40x magnification) detail the presence of red blood cells in the tunica media, with an infiltrate of leukocytes and fibroblasts.

**Fig. 2:**
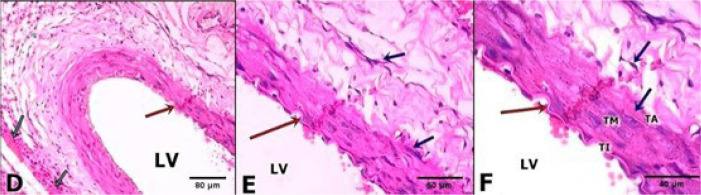
D, E and F belonging to the Chitosan Group (ChG) In D (20x magnification), a discrete hematic infiltrate is observed in the vascular wall, as well as a quantity of leukocytes and fibroblasts, in a more preserved tissue area. Details in E (40x) and F (60x) with clear division of the tunic intima (TI), media (TM) and adventitia (TA), closer to the great support of collagen fibres and elastic fibres. Note green arrows in D indicate the presence of degraded remnants of chitosan.

**Fig. 3:**
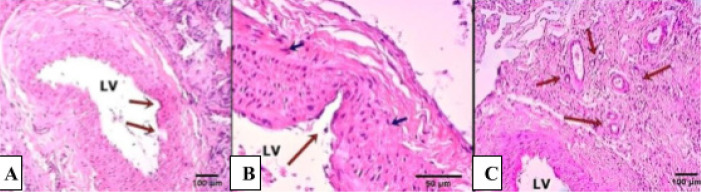
Photomicrographs of the region of surgical specimens extracted from samples of the TachoSil® Group 7 days after the operation, stained with haematoxylin and eosin. The arrows in A (10× magnification) point to discontinuity of the vascular wall with a 40× increase in B. The presence of leukocytes and fibroblasts can also be observed. Note, through the indication of the arrows in C (10× magnification), the discreet presence of microcirculation, possibly originating from the neovascularization process. LV=lumen of the vessel.

**Fig. 4:**
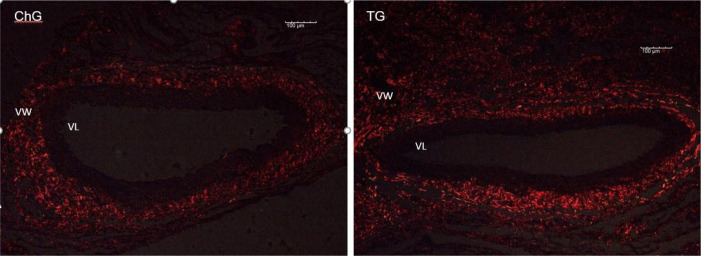
Photomicrograph of surgical specimens collected from the experimental chitosan group (ChG) and TachoSil® group (TG) on the 21^st^ postoperative days. Specimens stained with Picrosirius Red. Note the presence of collagen fibres adjacent to the vessel walls (VW) and surrounding the lumen of the vessel (VL). The intensity and birefringence of red characterize the presence of type I fibres. Type III collagen fibres were not significantly identified.

Arterial fragments of the areas that underwent intervention were removed and prepared for histological study with haematoxylin and eosin (Figures 1, 2, and 3) and with Picrosirius Red (Figure 4).

Nikon Eclipse E200 photomicroscope was used (4×, 10×, 20×, 40×, and up to 60× magnification objectives) as well as the Motic software for digital histological analysis and data recording through light microscopy. The samples stained with Picrosirius Red were submitted to polarized light microscopy to evaluate the birefringence of collagen fibres. Each histological section was examined to grade the inflammatory response using the modified Ehrlich-Hunt^[5]^ numerical scale that takes into account the presence of erythrocytes, leukocytes, macrophages, fibroblasts, neovascularization, and the occurrence of necrosis, as shown: 0, no evidence; 1, occasional occurrence; 2, slight dispersion; 3, abundant evidence; and 4, confluence of cells or fibres.

### Statistical Analysis

For statistical analysis, the IBM Corp. Released 2017, IBM SPSS Statistics for Windows, Version 25.0, Armonk, NY: IBM Corp. was used, and the chi-squared test was performed. To compare two independent variables, Mann-Whitney nonparametric test was used, and to three or more independent variables, Kruskal-Wallis nonparametric test was used.

## RESULTS

There was no significant difference in relation to the mass of the animals between the groups, thus the groups were homogeneous.

### Haemostatic Capacity

For analysis of the haemostatic capacity, the time spent until the haemorrhage ceased and the volume of blood lost were evaluated.

The time needed to suppress haemorrhage (time to haemostasis) was significantly longer in the CG when compared to the experimental groups (*P*≤0.001). Although the time to haemostasis in the TG was shorter when compared to that of the ChG, this difference was not significant (*P*=0.073) (Table 1).

**Table 1:** Time required to achieve haemostasis by group.

Group	Average*(s)	Median(s)	*P*-value
CG (n=16)	181.25±67.49	170.00	0.001
ChG (n=16)	82.50±33.66	70.00	
TG (n=16)	65.63±39.49	47.50	
Comparing ChG and TG			
ChG (n=16)	82.50±33.66	70.00	0.73
TG (n=16)	65.63±39.49	47.50	

CG=control group; ChG=chitosan group; TG=TachoSil® group

* Values expressed as mean ± standard deviation Comparing ChG and TG with CG, *P*<0.001 n=16 means that 1 procedure was done in each common femoral artery of the 8 animals

The groups were homogeneous in relation to the blood volume of the animals. The relative blood loss during and after the procedure was significantly lower (*P*<0.05) in the experimental groups when compared to the CG: using the Mann-Whitney nonparametric test, the *P*-value was 0.02 when comparing the CG *versus* the experimental groups (ChG and TG). However, when comparing only the experimental groups (ChG *versus* TG), the *P*-value was equal to 0.18, being greater than the significance level set at *P*>0.05. The results are shown in Table 2.

**Table 2:** Blood volume lost during haemorrhage in grams.

Group	Average*(g)	Median(g)	*P*-value
CG (n=16)	0.26±0.07	0.26	0.025
ChG (n=16)	0.19±0.17	0.15	
TG (n=16)	0.11±0.09	0.04	
Comparing ChG and TG			
ChG (n=16)	0.19 ± 0.15	0.15	0.18
TG (n=16)	0.11 ± 0.09	0.04	

CG=control group; ChG=chitosan group; TG=TachoSil® group

* Values are expressed as mean ± standard deviation Comparing ChG and TG with CG, *P*=0.025 (Kruskal-Wallis test) n=16 means that 1 procedure was done in each common femoral artery of the 8 animals

### Macroscopic Analysis

The examination of the surgical wound showed that in all animals there was adequate healing and there were no signs of infection.

Using the Kruskal-Wallis nonparametric test, it was found that there was no significant difference between the degree of tissue adhesions between the groups (Table 3).

**Table 3:** Classification of adhesions according to the classification by Knightly et al.^[4]^.

Group	Average*	Median	*P*-value**
CG (n=16)	0.50±0.33	0.50	0.135
ChG (n=16)	1.00±0.63	1.00	0.122
TG (n=16)	0.56±0.32	0.50	0.667

CG=control group; ChG=chitosan group; TG=TachoSil® group

* Values are expressed as mean ± standard deviation, followed by the median

** *P*=Kruskal-Wallis test

### Microscopic Analysis

Histological findings demonstrated that the healing process followed the usual healing characteristics at seven and 21 days after the procedure in the three groups. In the slides of the animals euthanized at seven days, there was a predominance of red blood cells and leukocytes, while in the slides of the animals euthanized at 21 days, there was a predominance of neovessels and fibroblasts.

Considering the time of 21 days from the date of the procedure, significant differences were observed in four of the six items analysed: greater number of red blood cells (*P*≤0.001), leukocytes (*P*≤0.001), neovessels (*P*≥0.003), and fibroblasts (*P*≤0.032). The presence of macrophages was very rare, and no giant cells or areas of fibrosis were found. The values of the findings are shown in Table 4.

**Table 4:** Microscopic analysis according to the modified Ehrlich-Hunt classification^[5]^.

	CG (n=16)	ChG (n=16)	TG (n=16)	*P*-value**
Red cells	3.75±0.46*	0.56±0.73	0.25±0.45	0.001
Leukocytes	2.75±1.03*	0.38±0.31	0.56±0.51	0.001
Neovascularization	2.25±1.39*	0.88±0.91	0.31±0.60	0.003
Fibroblasts	2.63±0.74*	1.38±1.02	1.69±1.14	0.032

CG=control group; ChG=chitosan group; TG=TachoSil® group

* Values are expressed as mean ± standard deviation, followed by the median

** Kruskal-Wallis test n=16 means that 1 procedure was done in each common femoral artery of 8 the animals

## DISCUSSION

Haemostasis is a primary condition for preventing complications inherent to surgical procedures^[6]^. Uncontrolled haemorrhage can prolong hospital stay, increase costs, and can lead to hypovolemic shock, failure of the cardiovascular system, and, consequently, death. Among the spectrum of substances available to help prevent this unwanted haemorrhage, natural and biodegradable polymers, such as chitosan, can lead to better results than synthetic polymers^[7]^.

When chitosan comes in direct contact with the blood, blood clots start to form due to the attraction of erythrocytes. The mechanism of action is based on an ionic interaction between positively charged chitosan acetate and negatively charged red blood cells. Deficiencies in the coagulation process do not seem to interfere with the coagulation process fostered by the application of chitosan, since the mechanism of action of chitosan is not in the coagulation cascade^[8]^.

### Haemostatic Capacity

The present study demonstrated that the use of chitosan membrane as well as the collagen sponge showed significant differences (*P*<0.05) in the measurement of the time until reaching haemostasis, when compared to the CG. The effectiveness of the chitosan membrane is explained by the interaction of the chitosan NH3 + ammonia groups with red blood cells. Platelets, which have a negative electrical charge, join chitosan through an ionic bond, which results in the formation of a thrombus, due to platelet aggregation, inducing haemostasis^[9]^.

This result is consistent with the prospective controlled and randomized study by Kang et al.^[6]^ in patients undergoing transradial approach and coronary interventions, in which it was demonstrated that the application of small chitosan-based blocks, combined with local pressure, obtained superior results when compared with the isolated application of local pressure. About 70% of patients in the study groups achieved haemostasis in the first 10 minutes, while 75% of patients in the CG (the isolated local compression group), took > 11 minutes to achieve haemostasis.

Although the CG showed a slightly longer time to stop bleeding than the TG, the difference between these two groups was not significant (*P*>0.05), demonstrating that both materials have similar haemostatic capacities.

Li et al.^[10]^ developed microspheres composed of chitosan through chemical processes known as microemulsion and thermally-induced phase separation. The authors compared the microspheres developed with haemostatics of commercially used chitosan, such as Celox® compresses, from ChitoFlex®, TraumaStat® linings, and bandages from HemConTM, in order to evaluate and compare the ability of these materials to promote haemostasis. It was analyzed the *in vivo* haemostatic ability of the materials to suppress haemorrhage caused by amputation of the tail of rats and by liver laceration. Both the time until haemostasis and the lost blood volume were measured. The chitosan microspheres developed by Li et al.^[10]^ showed better results, that is, less time to reach haemostasis and less blood volume lost due to bleeding. In the Li et al.^[10]^ study, while blood loss in the CG was 1.450 g±0.523 g and in the chitosan compound group it was 0.273 g±0.174 g, the amount of blood lost in the group of the smaller and denser chitosan microspheres was 0.061 g±0.034 g.

Misgav et al.^[11]^ highlighted in their study the importance of bleeding time in chronic haemodialysis-dependent patients. According to them, haemorrhage in this type of patient can easily exceed 45 minutes, even with continuous direct pressure with gauze pads at the puncture site. However, prolonged and repetitive compression can induce the formation of a thrombus and eventually lead to the occlusion of some blood vessel, generating serious complications. Misgav et al.^[11]^ compared the efficacy of chitosan acetate compresses with conventional gauze pads in 15 chronic haemodialysis patients with prolonged bleeding at the puncture site and found that chitosan-based pads obtained significantly better results than conventional gauze pads (*P*<0.01).

### Tissue Adherence

Tissue adhesion was analysed in CG21, ChG21, and TG21 groups. The results expressed in the adherence table suggest that both the chitosan membrane and the collagen and fibrinogen sponge did not cause a scar exacerbation. In the study by Zhou^[12]^ with chitosan compound gels, it was found a significant reduction in tissue adherence levels when compared to the CG. Although in the present study the CG also showed higher levels in the classification of adhesions, the connective tissues in general were slightly adhered to the adjacent tissues, not revealing a significant difference.

### Local Inflammatory Reactions

Histopathological examination showed that red blood cells, leukocytes, neovascularization, and fibroblasts were more pronounced in the CG than in other experimental groups. However, when ChG and TG methods were compared separately, using the Mann-Whitney test, there were no significant differences according to the modified Ehrlich-Hunt classification^[5]^.

The CG showed significantly more extravasation of erythrocytes in the vascular wall and in the surrounding areas (CG7 grade 4 = 100%; CG21 grade 3 = 50% and grade 4 = 50%). This fact may be related to the longer bleeding time and greater volume of blood loss presented by this same group. Although the TG had the lowest classification of red blood cell concentration (TG7 grade 0 = 62.5%, grade 1 = 37.5%; and TG21 grade 0 = 87.5%, grade 1 = 12.5%), the difference from ChG (ChG7 grade 0 = 37.5%, grade 1 = 37.5%, grade 2 = 25%; and ChG21 grade 0 = 75%, grade 1 = 25%) was not significant (*P*>0.05). The extravasation of red blood cells to the vascular wall and surrounding areas is directly related to the haemostatic capacity of the materials discussed before, reinforcing the haemostatic potential of the chitosan membrane.

As mentioned, after the epithelial and vascular tissues have suffered the injury and the platelet plug has formed, the leukocytes, mainly neutrophils, begin to migrate to the injury site to continue the healing process. Leukocyte infiltrates were observed in all groups. The group with most leukocyte activity was CG (CG7 grade 1 = 25%, grade 3 = 25%, grade 4 = 50%; and CG21 grade 2 = 50% and grade 3 = 50%). Thus, there was a significant difference (*P*<0.05) when compared to ChG and TG. ChG was the group that obtained the most discrete leukocyte infiltration of the three groups (grade 0 = 62.5% and grade 1 = 37.5% for the sum of ChG7 and ChG21), although there was also no significant difference in the presence of leukocytes when compared to TG.

In the three groups (CG, ChG, and TG), the samples taken from the animals euthanized on the 7^th^ postoperative day showed more leukocyte infiltrates (CG grade 1 = 25%, grades 3 and 4 = 50%; ChG grade 1 = 62.5 %; and TG grade 1 = 75%) than those from animals euthanized on the 21^st^ postoperative day (CG grades 2 and 3 = 50%; ChG grade 1 = 12.5%; and TG grade 1 = 37.5%). These observations were expected since the groups submitted to euthanasia on the 7th postoperative day are chronologically closer to the end of the late inflammatory phase and the beginning of the proliferative phase than the groups submitted to euthanasia on the 21^st^ postoperative day.

Acting in the final stages of the coagulation cascade, the sponge composed of collagen, fibrinogen, and thrombin accelerates the healing process. There was an improvement in helping in the efficiency of the coagulation process, and, consequently, a reducing of the harmful effects of the lesion, and mitigation the inflammatory reactions necessary in the process of healing.

Previous studies^[13,14]^ have shown that chitosan increases the capacity of inflammatory agents, such as neutrophils and macrophages, in addition to acting in all stages of healing. Furthermore, it has been reported that the chitosan haemostatic activity in the inflammatory phase can increase the healing process, accelerating the migration of leukocytes to the lesion site. Since the inflammatory phase is present between the first 24 and 72 hours, the acceleration of the migration of these inflammatory agents would lead to the early elimination of foreign substances. This, in turn, advances the process of fibroplasia and reepithelization and, consequently, the healing process itself^[13,14]^. Corroborating this hypothesis, Alemdaroğlu et al.^[15]^ found a shorter time in the process of repairing wounds caused by burns in the groups that used chitosan gels^[15-20]^.

The reduced leukocyte infiltration in samples that received the polymeric chitosan membrane may also be related to the antimicrobial properties reported in studies with chitosan compounds^[21]^.

Influenced by molecular weight and by the level of deacetylation, chitosan molecules are able to inhibit protein and messenger ribonucleic acid synthesis with the penetration of chitosan into the nucleus of microorganisms. In addition, it forms an external barrier, leading to the suppression of essential nutrients for growth and development of pre-existing microorganisms. Zheng et al.^[22]^ demonstrated that the antimicrobial effect of chitosan increases proportionally to the increase in chitosan concentration. A concentration of 1.0% had a rate of 100% inhibition of the growth of *Escherichia coli* and *Staphylococcus aureus* bacteria. An eventual inhibition of the grown of microorganisms at the lesion site induced by the properties of the chitosan membrane may have facilitated the inflammatory process, which is characterized by the reduction of the presence of leukocytes at the lesion site in the proliferative phase.

Karahaliloğlu et al.^[23]^ developed haemostatic sponges with a porous layer made up of chitosan, cellulose, and some active agents in the coagulation cascade, such as vitamin K and protamine sulphate, and analysed their performance in rat haemorrhages, also caused by injuries to femoral arteries. In the histological analysis carried out from resections of the arteries that received the materials of the experiment, although varying levels of inflammation were observed, the arterial walls remained intact. There were no responses with marked leukocyte infiltrates, corroborating the hypothesis that compounds of chitosan can be used safely to treat haemorrhagic lesions^[23,24]^.

When an injury occurs, fibroblasts are present at the beginning of the proliferative phase until the final stages of the healing process. CG was the group that most presented fibroblasts: 75% of the samples had grade 3 in the count of these cells. While in ChG and TG, 25% and 37.5% had grade 3, respectively. There was an increase in the fibroblast count in the 21-day groups and, consequently, an increase in the deposition of collagen and extracellular matrix. For example, 12.5% of the TG7 subgroup had the highest rating (grade 3), while 62.5% of TG21 had the same rating, practically five times more than TG7.

The increased proliferation of fibroblasts in the samples collected on the 21^st^ postoperative day when compared to the samples collected on the 7^th^ day may be related to the increased migration of fibroblasts to the lesion site, starting between seven and 14 days. At 21 days, the proliferative phase is ending, and the healing process is starting the remodeling phase, in which fibroblasts continue the synthesis of substances which had started in the previous phase. This happens in order to restructure provisional tissues, restore density, and strength tension and other characteristics that the vascular walls and adjacent tissues had before suffering the injury.

Howling et al.^[19]^ compared the ability of different levels of chitosan deacetylation to modulate fibroblast mitogenesis *in vivo*. The results obtained demonstrated that highly deacetylated chitosan polymers are better able to stimulate fibroblastic mitogenesis. Since chitosan used in the present experiment is highly deacetylated (75%-85%), the data converge for the efficiency of the fibroblasts present, due to the synthesis of the most organized and structured tissue.

Karahaliloğlu et al.^[23]^ analysed the fibroblast nucleus in tissues that received chitosan compounds as a haemostatic agent and found that the groups that were subjected to contact with chitosan polymers showed a larger nucleus, as well as shorter healing time. This observation highlights that the efficiency of the healing process and the restoration of the individual’s homeostasis, through the repair of injured tissues, do not depend exclusively on the quantity of inflammatory cells, but also on the performance of each cell, which can be directly influenced by the interaction with exogenous substances applied at the injury site.

In brief, the phenomenon of neovascularization is directly associated with the intensity of the inflammatory process and, consequently, with the participating structures, such as platelet by-products and neutrophilic proteins. Therefore, regarding the CG, the group with the most leukocyte infiltrates, it seems plausible that it was the group that also had the most newly formed blood vessels close to the injury site^[25]^.

The results obtained in this work show that the chitosan membrane showed haemostatic capacity and local inflammatory reaction similar to those of a commercially used haemostatic agent, the collagen membrane coated with fibrinogen and thrombin.

### Limitations

As in all experimental studies, extrapolation of the results to human procedures calls for evolution of data with further experimental and clinical trials.

## CONCLUSION

There were no significant differences between groups with respect to local tissue adherence. There were less exacerbated inflammatory reactions in animals that received chitosan membranes or collagen, fibrinogen, and thrombin sponge. The chitosan membrane showed haemostatic capacity similar to the collagen, fibrinogen, and thrombin sponge. Since chitosan is easy to find in nature, it has a positive effect to promote haemostasis and does not bring considerable local reactions, it might be used as a sealant in cardiovascular surgery.

**Table:** Authors’ Roles & Responsibilities

RNS	Substantial contributions to the design of the work; and the acquisition, analysis, and interpretation of data for the work; drafting the work and revising it
GBCC	Substantial contributions to the acquisition of data for the work; drafting the work
MRC	Substantial contributions to the analysis and interpretation of data for the work
EM	Substantial contributions to the analysis and interpretation of data for the work
MVHC	Substantial contributions to the conception and design of the work; and the analysis and interpretation of data for the work; drafting the work and revising it
